# Hepatitis C virus genotype frequency in Isfahan province of Iran: a descriptive cross-sectional study

**DOI:** 10.1186/1743-422X-7-69

**Published:** 2010-03-24

**Authors:** Sayyed H Zarkesh-Esfahani, Mohammad T Kardi, Masoud Edalati

**Affiliations:** 1 Department of Biology, Faculty of Sciences, University of Isfahan, Isfahan, 81746-73695, IR, IRAN; 2 Department of Immunology, Medical School, Isfahan University of Medical Sciences, Hezar Jerib Street, Isfahan, 81746-73695, IR, IRAN; 3 Department of Biology, Faculty of Sciences, University of Isfahan, Hezar Jerib Street, Isfahan, 81746-73695, IR, IRAN; 4 Department of Pathology, Medical School, Isfahan University of Medical Sciences, Hezar Jerib Street, Isfahan, 81746-73695, IR, IRAN

## Abstract

**Background:**

Hepatitis C is an infectious disease affecting the liver, caused by the hepatitis C virus (HCV). The hepatitis C virus is a small, enveloped, single-stranded, positive sense RNA virus with a large genetic heterogeneity. Isolates have been classified into at least eleven major genotypes, based on a nucleotide sequence divergence of 30-35%. Genotypes 1, 2 and 3 circulate around the world, while other genotypes are mainly restricted to determined geographical areas. Genotype determination of HCV is clinically valuable as it provides important information which can be used to determine the type and duration of therapy and to predict the outcome of the disease.

**Results:**

Plasma samples were collected from ninety seven HCV RNA positive patients admitted to two large medical laboratory centers in Isfahan province (Iran) from the years 2007 to 2009. Samples from patients were subjected to HCV genotype determination using a PCR based genotyping kit. The frequency of HCV genotypes was determined as follows: genotype 3a (61.2%), genotype 1a (29.5%), genotype 1b (5.1%), genotype 2 (2%) and mixed genotypes of 1a+3a (2%).

**Conclusion:**

Genotype 3a is the most frequent followed by the genotype 1a, genotype 1b and genotype 2 in Isfahan province, Iran.

## Background

Hepatitis C virus (HCV) is a small enveloped virus first isolated in 1989 and belongs to the family of flaviviridae [1]. Its genome is composed of a positive-sense, single-stranded RNA encoding a polyprotein comprising structural (core and envelope glycoproteins E1 and E2) and non-structural (NS2, NS3a/b, NS4a/b and NS5a/b) proteins. Acute HCV infection is often asymptomatic and approximately 70% of all cases progress to chronic hepatitis. This may lead to progressive liver disease, cirrhosis, liver failure and hepatocellular carcinoma within 20 to 30 years. Factors associated with disease progression following infection include the viral genotype, the patient's alcohol consumption and viral load [2]. Determination of the HCV genotype provides clinically important information that can be used to direct the type and duration of anti-viral therapy and to predict the likelihood of sustained HCV clearance after therapy [3,4]. Patients with HCV genotype 1 may benefit from a longer course of therapy and genotypes 2 and 3 are more likely to respond to a combination of interferon and Ribavirin therapy [5]. Most genotyping methods include a first step of reverse transcription (RT) of viral RNA and polymerase chain reaction (PCR) amplification. The conserved 5' non-coding region of the HCV genome has been used as a target for a number of diagnostic assays. This region can be characterized by probe hybridization or by variation in restriction patterns [6,7].

Global patterns for the distribution of different HCV genotypes are as follows:

1a is mostly found in North and South America, also common in Australia.

1b is mostly found in Europe and Asia.

2a is the most common genotype 2 in Japan and China.

2b is the most common genotype 2 in the US and north Europe.

2c is the most common genotype 2 in western and southern Europe.

3a is highly prevalent in Australia (40% of cases) and south Asia.

4a is highly prevalent in Egypt.

4c is highly prevalent in central Africa.

5a is highly prevalent only in south Africa.

6a is restricted to Hong Kong, Macau and Vietnam.

7a and 7b are common in Thailand.

8a, 8b and 9a are prevalent in Vietnam.

10a and 11a are found in Indonesia [8].

The aim of the present study is to determine the frequency of HCV genotypes in Isfahan province, Iran.

## Results

**Figure 1:**
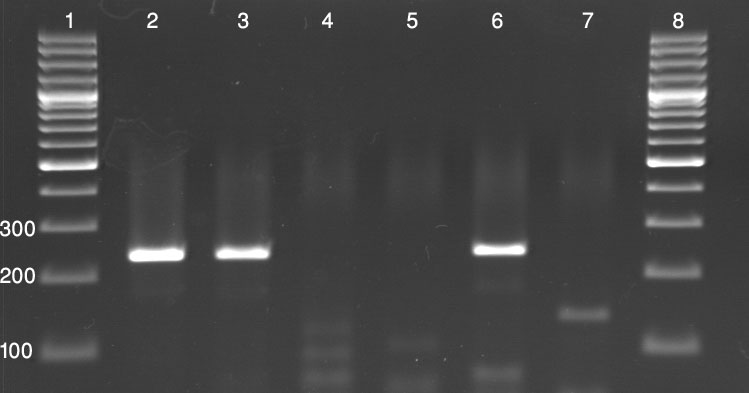
**A representative agarose gel electrophoresis of PCR products for the detection of HCV nucleic acid in plasma of patients positive for anti HCV antibodies**. Plasma samples from anti-HCV antibody positive patients were subjected to RT-PCR using general primers which are able to detect HCV nucleic acid by generating a 227 bp product. Lanes 1 and 8: 100 bp DNA size marker, lane 2: positive control, lanes 3 and 6: patients positive for HCV nucleic acid, lane 4: negative control, lanes 5 and 7: patients negative for HCV nucleic acid.

**Figure 2:**
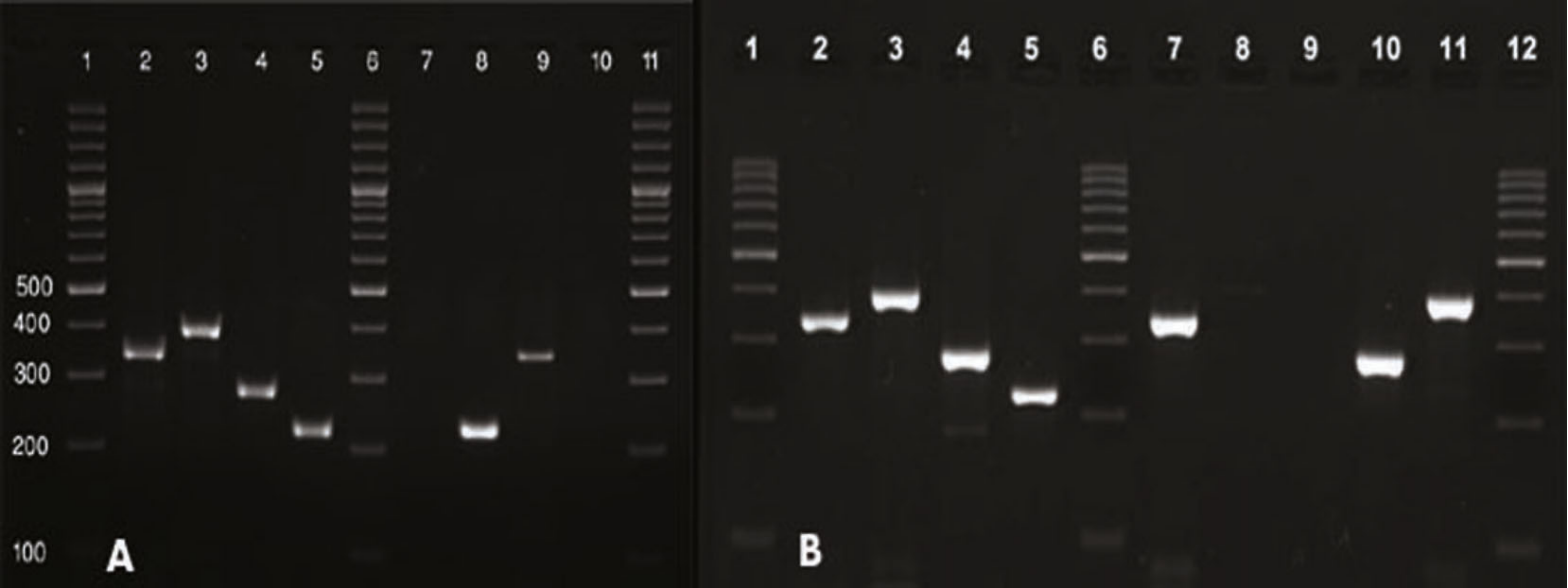
**Two representative agarose gels of PCR products demonstrating different HCV genotypes**. Plasma samples from patients positive for HCV nucleic acid were subjected to genotype determination using a specific RT-PCR kit which produces different size PCR products related to different genotypes. A) Lanes 1, 6 and 11: 100 bp DNA size marker, lane 2: positive control for genotype 1a (338 bp), lane 3: positive control for genotype 1b (395 bp), lane 4: positive control for genotype 2 (286 bp), lane 5: positive control for genotype 3a (227 bp), lanes 7 and 8: two sets of PCR amplifications for one patient resulting in genotype 3a, lanes 9 and 10: two sets of PCR amplifications for another patient resulting in genotype 1a. B) Lanes 1, 6 and 12: 100 bp DNA size marker, lane 2: positive control for genotype 1a (338 bp), lane 3: positive control for genotype 1b (395 bp), lane 4: positive control for genotype 2 (286 bp), lane 5: positive control for genotype 3a (227 bp), lanes 7 and 8: two sets of PCR amplifications for one patient resulting in genotype 1a, lane 9: empty well, lane 10: patient positive for genotype 2, lane 11: patients positive for genotype 1b.

**Figure 3:**
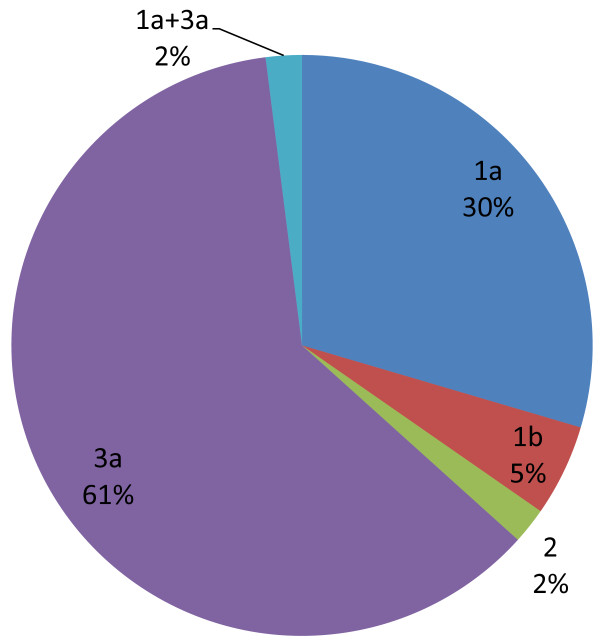
**Frequency of different HCV genotypes in Isfahan province of Iran**. HCV genotypes were determined in ninety-seven HCV RNA positive patients admitted to two large medical centers between 2007 and 2009 in Isfahan province, Iran. Predominant genotypes were: 3a (61.2%), 1a (29.5%) and 1b (5.1%). Less frequent was genotype 2 (2%). Mixed genotype infection of 1a and 3a was also observed in two cases (2%).

Patients were referred to two medical centers in Isfahan by specialists and were suspected of hepatitis. They were screened primarily for anti HCV antibodies. Samples from one hundred and forty-six patients who were positive for anti HCV antibodies were subjected to conventional RT-PCR for detection of HCV RNA (Figure 1). Ninety seven patients were identified positive for HCV nucleic acid. The mean age of the HCV nucleic acid positive patients was 35 years (16-78 years). Ninety-five subjects (97.9%) were male and two (2.1%) were female. Samples from these HCV RNA positive patients were used in a genotype determining method based on PCR technique. In this method different genotypes generate PCR products which are different in sizes (Figure 2). Genotypes of HCV were determined in all ninety seven patients. Genotypes 1a, 1b, 2 and 3a were detected and in some cases mixed infections with more than one genotype (1a+3a) were revealed. Predominant genotypes were genotype 3a (61.2%), 1a (29.5%) and 1b (5.1%). A less frequent genotype was genotype 2 (2%). There were also two cases with mixed genotype infection of 1a and 3a (2%) (Figure 3).

## Discussion

Hepatitis C infection may lead to a substantial health and economic burden over the next 10 to 20 years. From a public health perspective, the implementation of molecular tests as an integral part of the diagnostic and therapeutic management of infections with HCV should be imperative. Due to different treatment schedules between genotype 1 (48 weeks) and genotypes 2 and 3 (24 weeks), genotype distribution has a significant influence on the total costs and morbidity of HCV treatment. Nucleotide sequence analysis may be regarded as the gold standard for the identification of different HCV genotypes and subtypes, but it is generally believed to be impractical for routine clinical laboratory settings. Several alternative HCV genotyping procedures have therefore been suggested which are usually based on the analysis of PCR products by hybridization with genotype-specific probes or restriction length polymorphism analysis. In a routine diagnostic virology laboratory, simpler, faster and less expensive methods are required. In this study, we used a commercial RT-PCR genotyping kit which is able to determine common HCV genotypes in less than a few hours. This investigation was performed in Isfahan province, one of the most populated provinces, of Iran. HCV genotype 3a was found to be the most prevalent (61.2%), followed by genotype 1a (29.5%), 1b (5.1%), genotype 2 (2%) and mixed genotypes of 1a plus 3a (2%). The genotypes of all ninety-seven HCV nucleic acid positive patients were revealed by this kit. Although the kit used in this study was able to detect only genotypes 1a, 1b, 2 and 3a, all the samples were identified suggesting that the common genotypes are those reported here. We cannot rule out the possibility of mix infection with genotypes which the kit is not designed to detect, though according to the literature, other genotypes in that area are very rare.

There are several reports regarding the distribution of different HCV genotypes in different parts of Iran. In a population-based study 116 samples from HCV RNA positive patients from different parts of Iran were analyzed using PCR-RFLP. The authors reported that 1a, 3a, and 1b were the predominant genotypes with an overall prevalence rate of 61.2%, 25% and 13.8% respectively [9]. In a study in Fars province (Iran), during 2004-2005, among 188 subjects with HCV infection, 44.1% were genotype la, 42.0% genotype 3a and 13.8% genotype 1b [10]. They used Nested-PCR and PCR-RFLP for genotyping and stated that genotypes other than those reported could not be found in that area. In comparison with studies made in Iran's neighbor countries, it can be understood that the most common genotype in Kuwait, Iraq, and Saudi Arabia is type 4 [11]. However, genotype 1b in Turkey in the western border of Iran and genotype 3a in Pakistan in the eastern border of Iran are more prevalent [12,13]. Although genotype 4 is found almost exclusively in Middle East and western countries [14], this genotype is very uncommon in Iran. From different studies conducted in different parts of Iran, it generally could be concluded that genotypes 1a and 3a are the most prevalent in Iran while 1b and 2 are less frequent and other genotypes are rare [9,15]. A similarity has been reported between Iran and both Pakistan and India, in which genotype 3 is very prevalent and genotype 2 is rare [16,17]. It could be due to the high rate of immigration from these countries to Iran, especially when considering the fact that the prevalence of HCV infection in these countries is higher than in Iran. Genotypes 3a and 1a are more prevalent in intra-venous drug abusers (IVDU) in Europe and USA [18-20]. In the present study, 85 patients (86.5%) were IV drug abusers among whom HCV genotype 3a and 1a were more prevalent. It seems that there is a high similarity between the pattern of genotype in IVDU in Europe, the United States and Iran.

## Conclusion

In conclusion, our results are in accordance with other reports demonstrating the predominance of genotypes 3a and 1a and a very low frequency of genotype 2 in Iran which is different from Europe, USA and even some parts of Asia [11,21-24].

## Methods

### Patients and study design

This is a descriptive cross-sectional study. Patients who were suspected of hepatitis were referred to two large medical centers by specialists in Isfahan province (Iran) between March 2007 and April 2009. They were screened primarily for anti-HCV antibodies using a commercial ELISA kit. Those who were positive for anti-HCV antibodies were selected for HCV RNA detection using the RT-PCR technique. Out of 146 anti-HCV antibody positive patients, 97 were positive for HCV RNA. Patients who were positive for anti-HCV antibodies but were negative for HCV RNA were under treatment and were excluded. All samples from HCV RNA positive patients were selected for the study and were subjected to HCV genotype determination using commercial RT-PCR based kit. A waiver of consent was provided by the executive ethics committee of the medical center, as the samples used in this study were surplus to requirements following diagnostic investigations.

### Plasma samples

Ten ml venous blood samples were collected from each patient referred to the laboratory. Blood samples were collected in tubes containing EDTA. Centrifuged and separated plasmas were immediately stored at -80°C. To avoid RNA degradation, aliquots were not thawed more than once prior to analysis.

### HCV RNA detection

Plasma samples were tested for the presence of HCV nucleic acid using, with general primers, the conventional RT-PCR commercial kit (Qiagen, Germany) which is able to detect all different HCV genotypes. Tests were conducted according to manufacturer's recommendations. Briefly they were as follows: 50 μl plasma was added to 5.6 μl RNA carrier and 560 μl of lysis buffer, incubated at room temperature for 10 min before having 560 μl of ethanol (96-100%) added. 630 μl of the solution was transferred to the Minispin column, centrifuged at 6000 g for 1 min and then the spin column was placed into a clean 2 ml collection tube, the tube containing the filtrate being discarded. Subsequently 500 μl of washing buffer was added and centrifuged at 6000 g for 1 min, again the tube containing the filtrate was discarded. 500 μl of elution buffer was added and centrifuged at full speed for 3 min. Finally, 60 μl of dissolving buffer was added to the last spin column to collect the RNA. Small aliquots of isolated RNA were tested to assess the quality and quantity of RNA using a spectrophotometer (Eppendorf, Germany). Two μg of RNA was reverse-transcribed into cDNA using a master mix of 5XRT mix, M-MLV reverse-transcribe enzyme, RNAse inhibitor, dNTPs and ddH_2_O and then placed on a thermal cycler (Eppendorf, Germany) at 37°C for 30 min. Five μl of cDNA was subjected to PCR amplifications using general primers included in the kit, which is able to amplify all different genotypes of HCV. The PCR program was as follows: initial denaturation at 95°C for 5 min, and then 45 cycles at 95°C for 30 sec, 57°C for 15 sec, 72°C for 30 sec and a final extension at 72°C for 5 min. PCR products were subjected to electrophoreses and separation on 2% agarose gel, being visualized under UV light after ethidium bromide staining.

### HCV genotyping

Samples positive for HCV RNA were subjected to HCV genotyping using a commercial kit (Sacace, Italy) according to manufacturer's recommendations. This kit is designed for the detection of genotypes 1a, 1b, 2, 3a (most common HCV genotypes in Iran) by generating different size PCR products. The specificity and sensitivity of the kit are 100% and 1000 viral particles per ml respectively according to the manufacturer. For each patient two sets of PCR amplifications were carried out in two separate tubes containing primers for either genotypes 1a+1b or genotypes 2+3a. Five μl of cDNA was subjected to PCR amplifications using two sets of mixed primers included in the kit. The PCR program was as follows: initial denaturation at 95°C for 5 min, and then 42 cycles of 95°C for 1 min, 68°C for 1 min, 72°C for 1 min and a final extension at 72°C for 10 min. Genotype 1a generates a 338 bp PCR product, 1b 395 bp, 2 286 bp and 3a 227 bp. PCR products were electrophoresed, separated by 2% agarose gel and visualized under UV light after ethidium bromide staining.

## Competing interests

The authors declare that they have no competing interests.

## Authors' contributions

SHZ supervised the experiments and contributed to data analysis and the preparation of the manuscript. MTK performed all the experiments and contributed to data analysis. MA contributed to data analysis and the preparation of the manuscript. All authors read and approved the final manuscript.
